# Status epilepticus: review on diagnosis, monitoring and treatment

**DOI:** 10.1590/0004-282X-ANP-2022-S113

**Published:** 2022-08-12

**Authors:** Lecio Figueira Pinto, João Paulo Santiago de Oliveira, Aston Marques Midon

**Affiliations:** 1 Universidade de São Paulo, Faculdade de Medicina, Hospital das Clínicas, Departamento de Neurologia, Grupo de Epilepsia, São Paulo SP, Brazil.; 2 Universidade de São Paulo, Faculdade de Medicina, Hospital das Clínicas, Departamento de Neurologia, São Paulo SP, Brazil.

**Keywords:** Status Epilepticus, Diagnosis, Monitoring, Electroencephalography, Therapeutics, Estado Epiléptico, Diagnóstico, Monitoramento, Eletroencefalografia, Terapêutica

## Abstract

Status epilepticus (SE) is a frequent neurological emergency associated with high morbidity and mortality. According to the new ILAE 2015 definition, SE results either from the failure of the mechanisms responsible for seizure termination or initiation, leading to abnormally prolonged seizures. The definition has different time points for convulsive, focal and absence SE. Time is brain. There are changes in synaptic receptors leading to a more proconvulsant state and increased risk of brain lesion and sequelae with long duration. Management of SE must include three pillars: stop seizures, stabilize patients to avoid secondary lesions and treat underlying causes. Convulsive SE is defined after 5 minutes and is a major emergency. Benzodiazepines are the initial treatment, and should be given fast and an adequate dose. Phenytoin/fosphenytoin, levetiracetam and valproic acid are evidence choices for second line treatment. If SE persists, anesthetic drugs are probably the best option for third line treatment, despite lack of evidence. Midazolam is usually the best initial choice and barbiturates should be considered for refractory cases. Nonconvulsive status epilepticus has a similar initial approach, with benzodiazepines and second line intravenous (IV) agents, but after that, aggressiveness should be balanced considering risk of lesion due to seizures and medical complications caused by aggressive treatment. Usually, the best approach is the use of sequential IV antiepileptic drugs (oral/tube are options if IV options are not available). EEG monitoring is crucial for diagnosis of nonconvulsive SE, after initial control of convulsive SE and treatment control. Institutional protocols are advised to improve care.

## RESUMO

O estado de mal epiléptico (EME) é uma emergência frequente, com alta morbi-mortalidade. Segundo nova definição da ILAE de 2015, EME acontece pela falha dos mecanismos responsáveis ​​pelo término ou início das crises, sendo anormalmente prolongadas. Pela definição existem diferentes tempos entre EME convulsivo, focal e ausência. Tempo é cérebro. Ocorrem alterações nos receptores sinápticos, levando estado mais pró-convulsivante, com aumento risco de lesão cerebral e sequelas. O manejo do EME deve incluir três pilares: parar a crise, estabilizar o paciente para evitar lesão secundária e tratar a etiologia. EME convulsivo é definido quando duração é maior que 5 minutos e trata-se grande emergência. Os benzodiazepínicos são o tratamento inicial, devem ser administrados rapidamente e na dose adequada. Fenitoína/fosfenitoína, levetiracetam e ácido valpróico são opções com evidência para tratamento de segunda linha. Se EME persistir, uso dos anestésicos é provavelmente a melhor opção como terceira linha tratamento, apesar da falta de evidências adequadas. O midazolam costuma ser a melhor escolha inicial e os barbitúricos devem ser considerados para casos refratários. O EME não convulsivo tem abordagem inicial semelhante, com benzodiazepínicos e agentes segunda linha, mas após, a agressividade deve ser equilibrada considerando risco de lesão pelas crises e complicações pelo tratamento agressivo. A abordagem sugerida é uso de fármacos IV sequenciais (via oral/tubo quando opções IV não disponíveis). A monitorização por EEG é fundamental para o diagnóstico do EME não convulsivo, após controle inicial EME convulsivo e para controle do tratamento. Protocolos institucionais são recomendados.

## INTRODUCTION AND CLASSIFICATION

Status epilepticus (SE) is one of the most common neurological emergencies and is associated with high morbidity and mortality, as high as 40% in refractory cases^1^ ^,^ ^2^. 

 In 2015 the International League Against Epilepsy Task Force provided a new definition, proposing that SE is a condition resulting either from the failure of the mechanisms responsible for seizure termination or from the initiation of mechanisms, which lead to abnormally, prolonged seizures (after time point T1). This condition can have long-term consequences (after time point T2), including neuronal death, neuronal injury, and alteration of neuronal networks, depending on the type and duration of seizures^3^.

**Figure 1.**
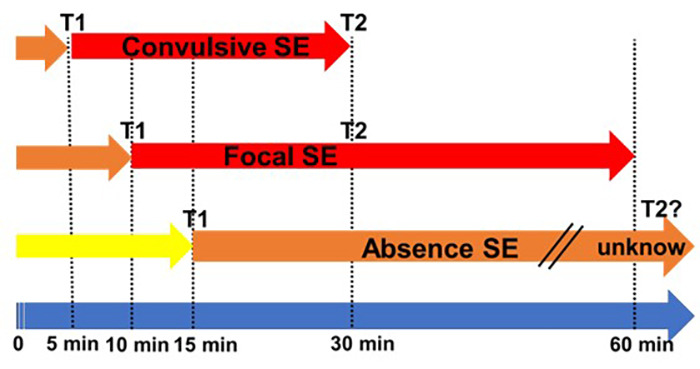
Different types of status epilepticus according to the duration.

Adapted from Trinka E et al. 2015^3^.

 As seen in the definition, there are two operational dimensions. The first (T1) is how long a seizure has to persist to be regarded as “continuous seizure activity” and by so, with a low chance of spontaneous termination. The second time point (T2) is when an ongoing seizure activity will put the person at risk of long-term consequences^3^. This is an important conceptual definition, because there are different forms of SE, with different risk and treatment strategies, that will be discussed in this review (Figure 1).

 The pathophysiology involved in SE suggests that there is a need for urgent diagnosis and treatment. Continued seizure activity has many consequences. There is receptor trafficking, and GABAergic receptors are endocytosed and have a decreased number in synaptic surface, leading to less response to treatment^4^. Also, glutamate receptors are upregulated in the synapse, leading to a more proconvulsant state. Synaptic plasticity altered gene expression, homeostatic failure and increased risk of sequelae and death are observed^5^. 

SE can be classified based on the semiology (Table 1), etiology, electroencephalography (EEG) correlates and age.

**Table 1.** Status epilepticus classification according to semiology.

With prominent motor symptoms
Convulsive SE (CSE, synonym: tonic-clonic SE); Myoclonic SE (prominent epileptic myoclonic jerks); Focal motor; Tonic status; Hyperkinetic SE.
Without prominent motor symptoms (i.e., nonconvulsive SE, NCSE)
NCSE with coma (so-called “subtle” SE); NCSE without coma; 2.2.1Generalized (absence status); 2.2.2 Focal (without impairment of consciousness - aura continua, with autonomic, sensory, visual, olfactory, gustatory, emotional/ psychic/experiential, or auditory symptoms -, aphasic status or with impaired consciousness); 2.2.3 Unknown whether focal or generalized.

Because of the differences in presentation and prognosis, treatment approaches differ between the different types of SE. The most frequent types of SE will be discussed in the following sections.

## CONVULSIVE SE

 Time is brain. Convulsive SE (CSE) is an emergency because most tonic-clonic seizures last less than two minutes^5^, so after five minutes treatment should be initiated because the chances of spontaneous cessation are low. Also, if CSE lasts more than 30 minutes there is compelling evidence of long-term sequelae and increased mortality^3^.

**Figure 2.**
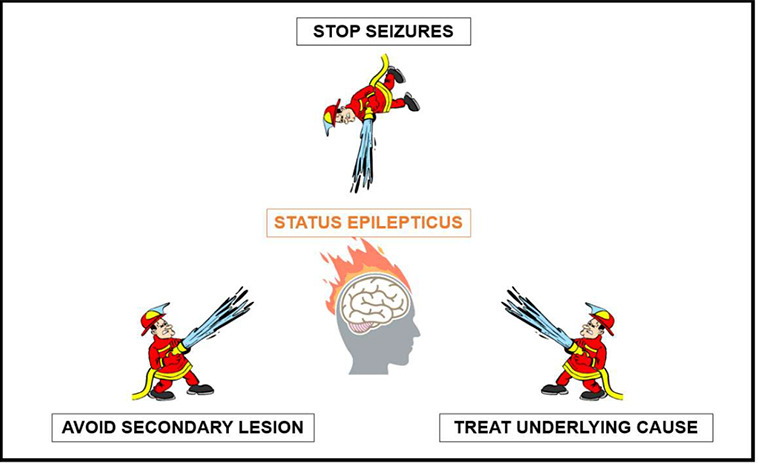
Pillars of status epilepticus treatment.

Therefore, treatment should be prompt, adequate and evidence-based, aiming at clinical and electroencephalographic cessation as quickly and safely as possible. Management of CSE must include three aspects: stop seizures, stabilize patients to avoid secondary lesions and treat underlying causes (Figure 2).

Treatment that is initiated early is much more likely to be effective^6^ and improve outcomes^7^. The first medication to be administered should be a benzodiazepine^8^, which should be started in pre-hospital care if possible. If there is no immediate venous access, the first option is intramuscular midazolam. When venous access is in place, intravenous diazepam (since there is no availability of intravenous lorazepam in Brazil) could be used as the first option of, and the dose can be repeated.

Respiratory and cardiac symptoms are the most common adverse events associated with IV anticonvulsant administration in adults with SE, however the rate of respiratory depression in patients with SE treated with benzodiazepines is lower than in patients with ongoing status epilepticus, indicating that these issues are an important consequence of untreated status epilepticus^8^.

Approximately 60 to 70% of patients with CSE will resolve with benzodiazepines^6^ ^,^ ^7^ ^,^ ^9^. Underdosing benzodiazepines is very frequent and is associated with failure to control and increased risk or refractoriness. 

When seizure continues, established SE is defined. Treatment should be done with an intravenous drug. There are the following options: phenytoin/fosphenytoin, valproate, levetiracetam, lacosamide and phenobarbital.

ESSET, a randomized, placebo controlled, double blinded multicenter trial evaluated levetiracetam, valproic acid and fosphenytoin for the treatment choices to treat established SE. They did not differ significantly with regard to effectiveness and safety^10^.

None of these options are available in Brazil, but fosphenytoin is a prodrug that needs to be converted into phenytoin to be active. At the end, time for conversion and brain penetration is expected to be the same. There is no clear evidence that it is more effective than phenytoin. 

Phenobarbital is an effective alternative, but with a worse adverse effect profile. It may be preferred in specific situations, such as alcohol withdrawal or to the drug itself (patients who discontinued use abruptly).

Lacosamide is an emerging option. No randomized controlled study in the context of CSE supports second-line use, but a series of cases suggest its efficacy.

According to ESSET^10^, almost half of patients (45-47%) will resolve SE with second line treatment. After treatment failure, no delay for escalation. If it is not possible to start anesthetic drug, to start another second line treatment could be an option, with the use of an available IV option such as levetiracetam, valproic acid, fosphenytoin. In Brazil the options would be lacosamide or phenobarbital.

Studies point that 31 to 55% of patients with established SE will not be controlled^11^ ^,^ ^12^. This stage is called refractory status epilepticus (RSE). Intravenous anesthetic drugs (thiopental/pentobarbital, midazolam or propofol) have been, and still are, the most commonly used options, despite there is no study providing Class I evidence for best option. Anesthetic selection is based on the advantages and side effect profile of each one, with consideration of each patient’s comorbidities and possible complications of the therapies. 

Currently, midazolam is the most commonly used drug for RSE due to faster onset of action, safety profile and short duration of effect^13^.

An interim analysis published in 2015 reported 488 cases from 44 different countries with RSE^14^. The most widely used anesthetic was midazolam (59%), followed by propofol (32%) and barbiturates (8%). SE was terminated in 74% of cases, but in most patients more than one anesthetic had to be administered to achieve this goal.

Propofol is used as first-line therapy in complex cases where pharmacokinetic properties are important and other drugs cause problematic hypotension. Propofol can cause a potentially fatal syndrome when given at high doses, known as the propofol infusion syndrome (PRIS). PRIS is characterized by cardiac and renal failure, metabolic acidosis, rhabdomyolysis, and enlarged or fatty liver. Risk factors include carbohydrate depletion, severe illness, mitochondrial dysfunction, and coadministration of catecholamines or steroids. To decrease the chances of developing PRIS, there is a suggestion to limit duration of administration to less than 48 hours and dose should not be higher than 4 mg/kg/hour^15^.

Thiopental/pentobarbital is preferred for severe cases. Increased mortality, prolonged mechanical ventilation, increased risk of infection are more common in barbituric coma^16^.

Recent papers suggest that Ketamine, a NMDAR inhibitor, should be considered in earlier phases. This drug has recently emerged as an alternative to traditional IV anesthetic agents. However, knowledge about ketamine and its potential usefulness is limited since it is often added to other continuous infusions. A meta-analysis of 110 adult patients revealed that ketamine may have helped control refractory status epilepticus in about 57% of patients^17^.

Anesthetic coma should be done for 24, in specific cases 48 hours, after SE control. EEG is necessary to confirm control and evaluate the depth of anesthesia. Some experts suggest a reduction of 25% of the dose every 6 hours, and EEG control is suggested to evaluate if electrographic SE recurs^18^. Two antiseizure medications, if possible intravenous, with adequate levels, should be administered before anesthetic reduction^19^.

If SE continues or recurs, it is suggested to restart a new cycle. New cycles should consider change to another anesthetic, associations such as midazolam and ketamine, midazolam and propofol. Use of thiopental/pentobarbital is suggested in these difficult cases. If the status continues to recur, the duration of individual cycles can be increased, but no good evidence to support that is available.

Before new withdrawals, consider association of three antiseizure medications. Again, they should be preferable intravenous, but nasogastric tube administration options (phenobarbital, lacosamide, levetiracetam, topiramate, valproic acid, perampanel, pregabalin, vigabatrin and brivaracetam) are acceptable, especially in places with limited access.

Prolonged anesthesia carries increasing iatrogenic risks, and a skilled intensive care unit (ICU) and monitoring for complications is mandatory. It is possible that in very refractory cases, with prolonged SE (weeks or months), risks associated with anesthetic use could outweigh the risk of brain lesion due to the pattern, and balance between these points should be done for treatment continuation and aggressiveness^18^.

**Figure 3.**
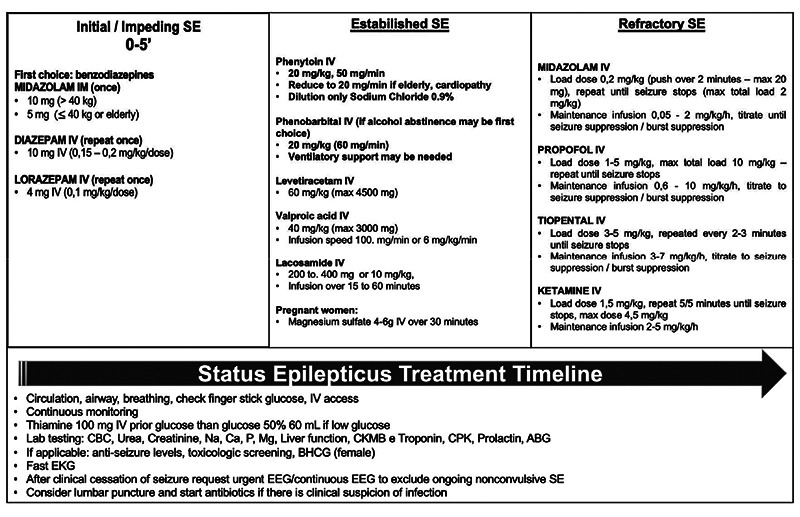
Status epilepticus treatment timeline.

SE: status epilepticus; IM: intramuscular; IV: intravenous; CBC: complete blood count; Na: sodium; Ca: calcium; Mg: magnesium; CKMB: creatine kinase myocardial band; CPK: creatine kinase; ABG: arterial blood gas; EKG: electrocardiogram; EEG: electroencephalogram.

SE control should be accessed with continuous EEG, to exclude nonconvulsive SE, nonconvulsive seizures and periodic patterns in the interictal-ictal continuum that warrant treatment^6^ ^,^ ^20^ (Figure 3). 

 Withdraw of care is not usually recommended in SE, because even after weeks of super refractory SE, some patients can recover with good functional status, especially when there are no clear or extensive lesions on neuroimage^19^.

In cases of super refractory SE other options could be considered: ketogenic diet^21^ ^,^ ^22^, surgery^23^ ^,^ ^24^, neuromodulation (VNS^25^ ^-^ ^27^, DBS ^28^ ^,^ ^29^, etc.), transcranial magnetic stimulation^30^ ^,^ ^31^. 

Intravenous pyridoxin is an effective treatment in the rare patients with an inborn error of metabolism of pyridoxin and isoniazid poisoning. Its efficacy has not been proven out of these situations, but some authors suggest a trial in super-refractory SE^18^.

Magnesium sulfate infusion is recommended for eclampsia^32^ and mitochondrial diseases such as POLG1. Its use has been suggested in super-refractory status epilepticus, although there is no clear evidence.

Cannabidiol is anecdotally described in case reports^33^.

Therapeutic hypothermia for treatment of refractory and super refractory SE was evaluated in the HYBERNATUS trial^34^, which failed to show benefits. At this moment, hypothermia is not recommended in this setting. 

Investigation is mandatory, especially in de novo SE. Etiology also had a considerable impact on the outcome. Outcome was linked to advanced age, etiology, new onset status epilepticus and NCSE^35^. Some tools have been suggested to help clinicians^36^ (Table 2).

**Table 2.** Investigations in status epilepticus.

Investigation	Descriptions
Basic investigations: in all patients	Computed tomography of brain Laboratory tests: blood glucose, renal and liver function tests, calcium, magnesium, drug levels Electrocardiogram Electroencephalogram
Other investigations to be considered, based on clinical history and examination	Magnetic resonance imaging (of brain) ± contrast Toxicology screen Infection screen, including uncommon infections: scrub typhus, mycoplasma pneumonia, HIV, syphilis, etc Cerebrospinal fluid for infection or encephalitis Antibodies for autoimmune encephalitis (blood and cerebrospinal fluid)^37^ Blood gases Thyroid function/antithyroglobulin and antiperoxidase antibodies Metabolic screen Vasculitis screen (ANA, dsDNA, Complement, ANCA, etc) Genetic investigation

Adapted from: Fung EL et al. 2017 ^38^.

ANA: antinuclear antibodies; dsDNA: double-stranded DNA; ANCA: anti-neutrophil cytoplasmic antibodies; HIV: human immunodeficiency virus.

NORSE (New-Onset Refractory Status Epilepticus) is not a specific diagnosis, it is a clinical presentation in patients without active epilepsy or relevant previous neurologic disorder, without a clear acute or active structural, toxic or metabolic cause for refractory SE. FIRES is a subcategory of NORSE, applicable for all ages, that requires a prior fever/febrile infection starting between 2 weeks and 24 hours prior to onset of refractory status epilepticus, with or without fever at onset^39^.

When etiology is known, autoimmune encephalitis is the most frequent cause of NORSE/FIRES. Studies suggest a better outcome with immunotherapies, especially when therapy is started early. Experts suggest starting treatment after 48-72h, with reasonable exclusion of infectious causes^40^. First-line treatments are steroids, intravenous immunoglobulins, and plasma exchange.

The main differential to consider when assessing patients with suspected (convulsive) status epilepticus are dissociative seizures, also known as functional or psychogenic non-epileptic seizures (PNES). In ESSET, 10% of randomized patients had a final diagnosis of PNES^10^. Prolonged PNES are reported by 78% of patients with PNES and lead to admissions to the intensive care unit (ICU) in 18%-27%^41^ ^,^ ^42^. Paola et al.^43^ has an interesting paper on semiology for bedside differentiation of ES and PNES. 

Institutional protocols are needed to guide diagnosis and treatment, with drug choices, flowcharts and staff training for this emergency situation^44^.

## NONCONVULSIVE SE

Nonconvulsive status epilepticus (NCSE) is, by definition, simply SE without prominent motor symptoms^3^ ^.^ The signs and symptoms of NCSE are broad and often subtle, including, although not limited to, inattention, disorientation, confusion, abulia, abnormal eye movements (e.g. gaze deviation or nystagmus), subtle repetitive facial or distal movements of extremities, and in more severe cases, stupor and coma^45^ ^-^ ^47^. EEG depicts different findings: focal or generalized findings, multiple seizures (including cyclic seizures) or a continuous pattern^20^ ^,^ ^48^. 

Nonconvulsive SE can occur in up to 10% of medical and surgical intensive care unit (ICU) patients in coma that undergo continuous EEG monitoring. After acute brain injury, prevalence of NCSE can be even higher^49^ ^,^ ^50^. 

### Clinical features of nonconvulsive status epilepticus^51^

- Altered mental status (82%): Confusion (49%), Coma (22%), Lethargy (21%) Memory loss (8%);
- Speech disturbance (15%);
- Myoclonus (13%);
- Unusual behavior (11%);
- Anxiety, agitation and delirium (8%);
- Extrapyramidal signs (7%);
- Hallucinations (6%).

NCSE is the most frequent type of SE, and it can be characterized by electrographic patterns lasting ≥ 10 minutes or present for ≥20% of the 60-minute recording. EEG is an essential tool for the diagnosis of NCSE^52^ because the clinical signs (if even present) are often subtle, unclear, or nonspecific. The Modified Salzburg Consensus is the most comprehensive diagnostic criterion, as it aggregates electroencephalographic findings, particularly the frequency and types of periodic patterns, with clinical findings, neuroimaging and toxic-metabolic disorder, with a sensitivity of 98% and specificity of 90%^53^.

The criteria^54^ ^,^ ^55^ state that either epileptiform discharges (EDs) or rhythmic delta activity (RDA) needs to be present to diagnose NCSE during 10 s at least. Depending on the frequencies shown in EDs or RDA, two ways to proceed with the diagnosis are presented:

1. EDs frequency >2.5 Hz: the patient is immediately diagnosed with NCSE.
2. EDs frequency ≤2.5 Hz or >0.5 Hz in RDA: at least one secondary criterion is needed.

- Spatiotemporal evolution;
- Subtle clinical ictal phenomena;
- Anti-seizure drug (ASD) trial, preferably intravenous, with a clinical and electrographical response).

### Possible NCSE is diagnosed if EDs frequency ≤2.5 Hz or >0.5 Hz in RDA with the following criterion

- fluctuation without criteria for evolution;
- ASD trial only with EEG response.

NCSE occurs in around 14% of patients after CSE treatment, with or without clinical manifestations^20^, and up to 48% have some type of epileptiform abnormality, usually periodic. EEG monitoring and treatment of these patterns has clinical relevance.

Prognosis is mainly associated with etiology. Comorbidities, age and duration also affect their severity, brain injury potential and prognosis^56^ ^-^ ^58^. 

However, there are a variety of rhythmic and periodic patterns with a high degree of uncertainty regarding their ictal nature. These present a diagnostic and therapeutic dilemma to electroencephalographers, intensivists, and general neurologists taking care of these patients. The term “ictal interictal continuum” (IIC) was first introduced by Pohlmann-Eden et al. in 1996 ^59^, who described periodic lateralized epileptiform discharges (formerly called PLEDs, now referred to LPDs according to the new ACNS nomenclature)^60^ as “an electrographic signature of a dynamic pathophysiological state in which unstable neurobiological processes create an ictal interictal continuum, with the nature of the underlying neuronal injury, the patient’s preexisting propensity to have seizures, and the coexistence of any acute metabolic derangements all contributing to whether seizures occur or not”^59^. 

The use of the term has now expanded to include other rhythmic and periodic patterns that are not definitely ictal, but could still be, and that may contribute to neuronal injury in certain clinical settings. There is no consensus agreement on IIC patterns definition, but these generally include:

- Periodic or rhythmic pattern with frequency ≥ 1 Hz but ≤ 2.5 Hz with duration ≥ 10 seconds;
- Periodic or rhythmic pattern with frequency ≥ 0.5 Hz but < 1 Hz with duration ≥ 10 seconds, associated with the plus modifier or fluctuation;
- Lateralized delta rhythmic activity at >1 Hz but ≤ 2.5 Hz with duration ≥ 10 seconds associated with plus modifier or fluctuation, but does not include the GRDA pattern;
- Patterns that cannot qualify as an electrographic seizure or SE.

Clinical and/or electroencephalographic seizures, periodic patterns, with a frequency greater than 2.5 Hz or, even when at a lower frequency, which have an associated plus modifier, evolution or fluctuation, which are part of the Ictal-Interictal Continuum and sometimes configure NCSE , are associated with hypermetabolism on PET-FDG^60^, increased intracranial pressure, brain temperature and cerebral oxygen perfusion partial pressure, configuring a “metabolic crisis” with a high lactate/pyruvate perfusion ratio, reduced brain glucose consumption, oxidative metabolism and impairment of oxidation and reduction mechanisms^61^. The potential for injury caused by IIC and NCSE has not yet been clearly defined, but evidence indicates that these changes can worsen or cause functional and/or structural injuries, especially in penumbra areas, accentuating deficits and delaying functional and consciousness recovery. Based on current knowledge, treatment of these patterns observed in the IIC and NCSE is recommended to reduce the risk of structural injuries and improve functional recovery^62^ ^-^ ^64^. 

**Figure 4.**
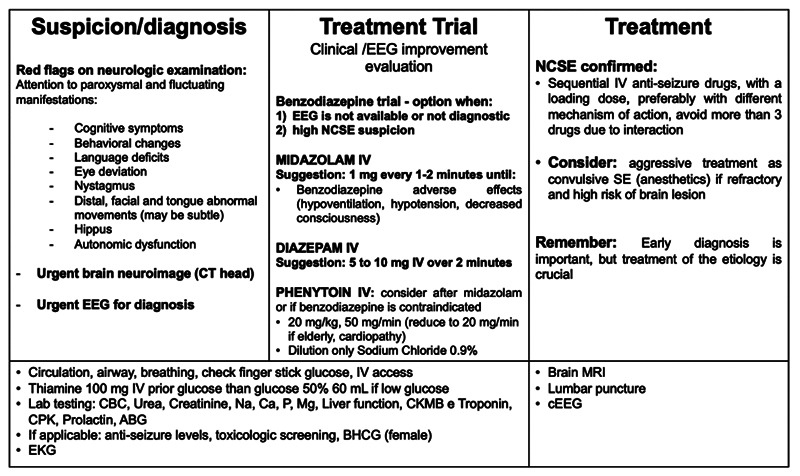
Nonconvulsive status epilepticus evaluation and treatment.

The goal of NCSE treatment is to control seizures and patterns in the IIC, limiting secondary injuries and dysfunction. However, this should be done gradually and with less intensity/aggressiveness than in the treatment of CSE^65^ (Figure 4).

Clinical findings (such as level and content of consciousness, subtle ictal phenomena), electrographic pattern and its evolution during continuous EEG, structural neuroimaging, functional neuroimaging (SPECT, PET) and, if available, other neuromonitoring parameters should be used to define treatment intensity, with the objective of controlling electrographic changes, balancing with medical complications caused by aggressive treatment.

Initial treatment with benzodiazepine plus a second line IV treatment are similar to CSE, but if seizures or IIC patterns persist, another anti-seizure drug, preferably IV, should be used. In some cases, tube/oral use is acceptable. Loading doses should be used to quickly obtain therapeutic levels, as well as maintenance doses according to the half-life of each ASD. We recommend sequential use of ASD, with attention to associations with different mechanisms of action and avoiding the association of more than 3 ASD. It is recommended to withhold medications that have not significantly impacted the control of seizures/patterns, to avoid interactions with ASD and other drugs in use.

The results of this approach should be evaluated preferably with continuous EEG, with the objective of controlling electrographic changes.

In severe, prolonged and refractory cases, especially when there is evidence of ongoing injury (structural or functional neuroimaging alteration, metabolic crisis evidenced by neuromonitoring), more aggressive treatment with anesthetic coma should be strongly considered.

In NCSE, anesthetic coma, when indicated, should be done for a limited time, with attention to complications such as hypotension, bacterial translocation, sepsis, cardiac depression. Prolonged and intense comas can cause more complications, injury and mortality than NCSE itself. Strategies to limit epileptogenesis such as reducing the “Seizure Burden” may be more appropriate.

Patients with super refractory and long-term NCSE should have continued treatment, especially in young patients, without severe comorbidities and able to withstand treatment, especially when no new lesion/progression is observed on neuroimaging.

## EEG MONITORING

Continuous EEG is essential for diagnosis^19^, classification and monitoring treatment (therapeutic response, seizure quantification, dose adjustment) of SE, also allowing the correlation of changes in consciousness, eye and pupil movements such as nystagmus and other atypical movements.

The indications for cEEG are outlined in Table 3. The guiding principles for these indications are multifactorial.

**Table 3.** Indications for cEEG in SE.

Recent clinical seizure or SE without return to baseline >10 min	Ongoing non-convulsive status despite cessation of motor activity 18-50 %
Coma, including post-cardiac arrest	Frequent non-convulsive seizures, 20-60 %
Epileptiform activity or periodic discharges on initial 30 min EEG	Risk of non-convulsive seizures, 40-60 %
Intracranial hemorrhage including TBI, SAH, ICH	Frequent non-convulsive seizures, 20-35 %
Suspected non-convulsive seizures in patients with altered mental status	Frequent non-convulsive seizures, 10-30 %

Adapted from Brophy et al.2012^19^.

EEG: electroencephalogram; ICH: intracranial hypertension; SAH: subarachnoid hemorrhage; TBI: traumatic brain injury.

In patients being treated with continuous infusion ASD and anesthetics, in which most or all clinical manifestations resolve, cEEG is the only way to assess if treatment was successful. The use of video monitoring in conjunction with cEEG in the ICU may aid EEG interpretation and help assess the presence of clinical behaviors accompanying the ictal EEG, despite no prospective studies have formally assessed efficacy of adding video to cEEG in the setting^19^.

Continuous EEG should be initiated within preferably one hour of suspected SE. Overall, 88% of patients had the first seizure detected within 24 hours of cEEG. However, this was dependent on the patient’s neurologic status. The first seizure was detected in the first 24 hours of recording in 95% of noncomatose patients but in only 80% of comatose patients. Longer duration of cEEG monitoring is needed in comatose patients^66^, at least 48 h^66^ ^-^ ^70^. cEEG should be kept during the AED weaning trials and at least 24 h after cessation of electrographic seizures^66^ ^,^ ^66^ ^,^ ^70^. 

In RSE a super refractory SE cEEG monitoring is crucial since the vast majority of seizures are non-convulsive. The EEG endpoints are controversial, and options include burst suppression, complete background suppression or seizure suppression^71^ ^-^ ^73^. Seizure control and burst suppression are the choices for most of the authors. Also, 35% to 41% of these critically ill patients have periodic and rhythmic patterns (PRPs) when monitored on cEEG^74^ ^,^ ^75^, and some of them lie on IIC and warrant additional treatment^76^. 

Despite increasing use of cEEG over the last years^60^ ^,^ ^77^, a recent study showed that only 0.3% of the critically ill population received cEEG, despite the evidence that patients had a decreased risk of in-hospital mortality with its use. 

The Standardized Terminology of EEG in Intensive Care of the American Clinical Neurophysiology Society (ACNS 2021)^60^ allows the characterization of graph elements, periodic electroencephalographic patterns and their frequencies and modifiers, being useful in the standardization of electroencephalographic reports and in the diagnostic criteria of NCSE. We recommend using the ACNS Standardized Terminology to analyze cEEG recordings.

EEG characteristics observed in the first hour of recording, added to some clinical data, allow estimating the risk of occurrence of epileptic seizures. The Risk Score of Epileptic Seizures in Hospitalized Patients (2HELPS2B) allows to guide seizure risk for patients on cEEG and the recording time necessary for an adequate diagnosis^78^.

The 2HELPS2B system combines 5 readily observable EEG features with a single factor from the patient history (any known history of seizure, remote or acute) to assign a score between 0 and 7:

- History of seizures: 1 point
- Frequency >2 Hz of periodic and rhythmic pattern: 1 point
- Sporadic epileptiform discharges: 1 point
- Presence of LPD(lateralized PD), BIPD (bilateral independent PD) or LRDA (lateralized rhythmic delta activity): 1 point
- Presence of “plus” features (+R, +F, +FR)
- Brief [ictal] rhythmic discharges: 2 points

The 2HELPS2B allows to guide the recording time necessary for an adequate diagnosis of epileptic seizures in patients at risk (Table 4) ^79^.

**Table 4.** Monitoring time with cEEG based on seizure risk.

Risk	2HELPS2B (pts)	Global risk	Seizures risk after adequate recording time (false negative)	Recommended registration duration
Low	0	3.1%	3.1%	1h
Intermediary	1	12.0%	4.0%	12h
High	≥2	26.6%	3.1%	≥24h

Adapted from Struck AF et al. 2020 ^81^.

Legend: cEEG - continuous eletectroencephalogram; pts - points; 2HELPS2B - acronym for The Risk Score of Epileptic Seizures in Hospitalized Patients

As a relevant point, in patients with intermediary and high risk, the maintenance or appearance of findings that increase the risk of seizures, especially those within the IIC, should lead to changes in treatment and a longer observation period, which should be individualized.

In coma patients with IIC patterns, a sensible approach would be to identify the electroencephalographic patterns most associated with epileptic seizures, proposed by Ruiz, A et al. 2017 ^75^ and the Modified Salzburg Consensus^80^ and the approach described below:

- Possibly non-ictal patterns (isolated epileptiform discharges, GRDA) → correction of seizure “facilitating” factors.
- Rhythmic and periodic patterns (LPD, LRDA, GPD, etc.) → reduce epileptogenesis with ASD; dosage can be adjusted or associated with another ASD according to continuous EEG response and changes observed in other exams and neuromonitoring.
- If the patterns in item 2 are accompanied by clinical manifestations, frequency ≥ 2 Hz, presence of modifiers, such as fast superimposed, lateralized activity, fluctuation → treat to “normalize” or at least reduce epileptogenicity of the patterns, focusing on improve the level of consciousness and associated manifestations. Anesthetic coma may be considered in this scenario.

Therapeutic testing with a benzodiazepine in patients with dubious patterns should be done to aid in the diagnosis. Electrographic improvement is only a dubious answer, being conclusive when there is an associated clinical response. In some cases, the clinical response may be slow, occurring after more than 24 hours, therefore a clear electrographic response should set the standard as NCSE as possible. We suggest preferential use of benzodiazepines, with other non-sedating ASD as an option, such as IV phenytoin, IV lacosamide, or other options by tube/oral when another IV option is not possible (valproate, levetiracetam, topiramate, vigabatrin are good options). In cases at risk of lowering consciousness requiring intubation and/or hypotension, the use of 1 mg IV midazolam, repeated sequentially according to clinical-EEG response and patient stability may be useful.

## FINAL MESSAGES

SE is a frequent neurological emergency and deserves attention to its treatment, with institutional protocols addressing the sequence, dosage and available antiseizure medications. Training of the team involved in the care of these patients is advised. 

The management of SE must include three pillars: stop seizures, stabilize patients to avoid secondary lesions and treat underlying causes.

Benzodiazepines are the first line of treatment and should be used fast and with adequate doses. Treatment delay and underdosing are frequent and lead to refractory SE.

In patients who persist with seizures, treatment with a second line intravenous antiseizure medication is recommended, and the choices are levetiracetam, valproic acid, fosphenytoin/phenytoin (only the last one is available for IV use in Brazil).

Refractory CSE (failure of first and second line) has less evidence for the treatment, but anesthetics are recommended. Midazolam is the best choice, and barbiturates should be used in severe cases because they carry more risk.

Nonconvulsive SE has initial treatment (first and second line) similar to convulsive SE, but after that, it’s recommended the use of sequential IV antiepileptic drugs (oral/tube are options if IV options are not available). Treatment aggressiveness should be balanced considering risk of lesion due to seizures and medical complications. Anesthetics should be reserved for more severe cases and used for a limited time.

EEG monitoring is crucial for diagnosis of nonconvulsive SE because the clinical manifestations are unspecific. Also, after initial control of convulsive SE there is a significant risk of ongoing electrographic seizures and/or patterns that warrant further treatment. During SE the treatment and withdraw, especially with anesthetics, monitoring deep of sedation and seizure control could only be done safely with EEG monitoring.
